# [2-Formyl-4-methyl-6-({2-[2-(4-nitro­benzyl­amino)­ethyl­amino]­ethyl­imino}­meth­yl)phenolato]nickel(II) perchlorate

**DOI:** 10.1107/S1600536812021058

**Published:** 2012-05-12

**Authors:** Yang Wang, Jia-Wei Mao, Hui-Ting Song, Hong Zhou

**Affiliations:** a Key Laboratory for Green Chemical Processes of the Ministry of Education, Wuhan Institute of Technology, Wuhan 430073, People’s Republic of China; b College of Chemistry and Molecular Sciences, Wuhan University, Wuhan 430072, People’s Republic of China

## Abstract

In the unsymmetrical title complex, [Ni(C_20_H_23_N_4_O_4_)]ClO_4_, the coordination geometry for the Ni^II^ atom can be described as square planar. The aromatic rings in the two ligands are almost vertical, with a dihedral angle of 85.3°. In the crystal, cations and anions are linked by weak C(N)—H⋯O hydrogen bonding.

## Related literature

For Schiff base complexes containing polynitro­gen ligands, see: Gao *et al.* (2002 ▶); Souza *et al.* (2009 ▶); Tsubomura *et al.* (2000 ▶) and for nickel–Schiff base complexes, see: Wu *et al.* (2011 ▶); Cheng *et al.* (2011 ▶); Wang *et al.* (2008 ▶). For the synthesis, see: Zhou *et al.* (2009 ▶). For the preparation of 2,6-diformyl-4-methyl­phenol, see: Long & Hendrickson (1983 ▶); Mandal *et al.* (1989 ▶) and for the preparation of *N* ^1^-(2-amino­eth­yl)-*N* ^2^-(4-nitro­benz­yl)ethane-1,2-diamine, see: Hu *et al.* (2011 ▶); Jian *et al.* (2004 ▶).
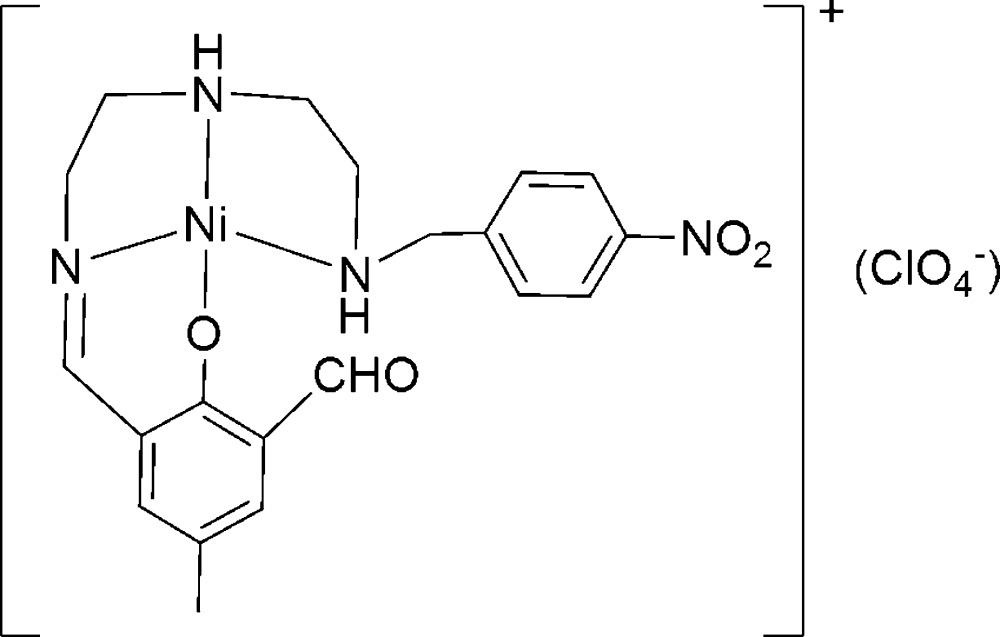


## Experimental

### 

#### Crystal data

[Ni(C_20_H_23_N_4_O_4_)]ClO_4_
*M* *_r_* = 541.58
Triclinic, 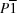

*a* = 9.5240 (14) Å
*b* = 9.6423 (14) Å
*c* = 14.024 (2) Å
α = 97.897 (2)°
β = 109.415 (3)°
γ = 107.453 (2)°
*V* = 1117.7 (3) Å^3^
*Z* = 2
Mo *K*α radiation
μ = 1.04 mm^−1^
*T* = 291 K
0.28 × 0.24 × 0.22 mm

#### Data collection

Bruker SMART APEX CCD diffractometer
Absorption correction: multi-scan (*SADABS*; Bruker, 2000 ▶) *T*_min_ = 0.739, *T*_max_ = 0.786
6276 measured reflections
4314 independent reflections
3004 reflections with *I* > 2σ(*I*)
*R*_int_ = 0.028

#### Refinement

*R*[*F* ^2^ > 2σ(*F* ^2^)] = 0.060
*wR*(*F* ^2^) = 0.138
*S* = 1.10
4314 reflections
308 parameters
H-atom parameters constrained
Δρ_max_ = 0.49 e Å^−3^
Δρ_min_ = −0.44 e Å^−3^

### 

Data collection: *SMART* (Bruker, 2000 ▶); cell refinement: *SAINT* (Bruker, 2000 ▶); data reduction: *SAINT*; program(s) used to solve structure: *SHELXTL* (Sheldrick, 2008 ▶); program(s) used to refine structure: *SHELXTL*; molecular graphics: *SHELXTL*; software used to prepare material for publication: *SHELXTL*.

## Supplementary Material

Crystal structure: contains datablock(s) global, I. DOI: 10.1107/S1600536812021058/kp2412sup1.cif

Structure factors: contains datablock(s) I. DOI: 10.1107/S1600536812021058/kp2412Isup2.hkl

Additional supplementary materials:  crystallographic information; 3D view; checkCIF report

## Figures and Tables

**Table 1:** Selected bond lengths (Å)

N2—Ni1	1.841 (3)
N3—Ni1	1.893 (3)
N4—Ni1	1.936 (4)
Ni1—O1	1.829 (3)

**Fig. 1.**
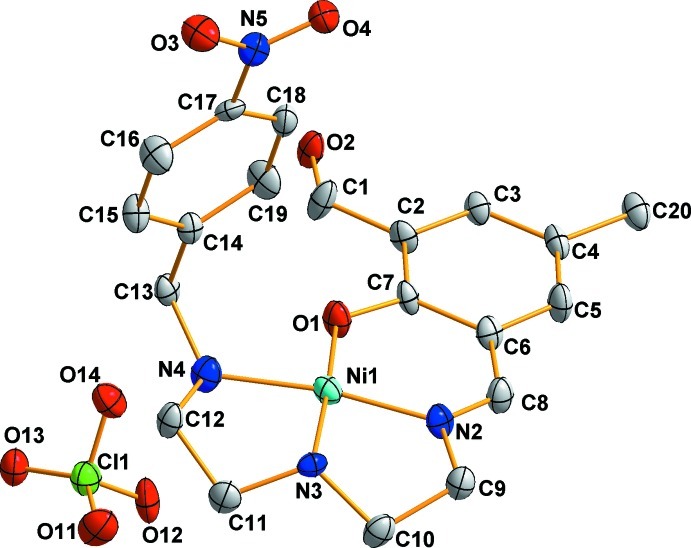
A view of the title complex with displacement ellipsoids at the 30% probability level. H atoms are excluded for clarity.

## supplementary crystallographic information

### Comment

For a long time Schiff base complexes containing polynitrogen ligands have been given considerable attention (Gao *et al.* 2002; Tsubomura *et al.* 2000; Souza *et al.* 2009). In this paper, we report on the synthesis and crystal structure determination of the title complex obtained by the reaction of 2,6-diformyl-4-methylphenol (*L^1^*) and the polynitrogen ligand N^1^-(2-aminoethyl)-N^2^-(4-nitrobenzyl)ethane-1,2-diamine (*L^2^*) in the presence of Ni(ClO_4_)_2_.6H_2_O.

In the title complex, [NiC_20_H_23_N_4_O_4_].ClO_4_, the Ni^II^ atom is four-coordinated by three amino N atoms from the ligand *L^2^* and one O atom from the ligand *L^1^*, the basal bond distances around the Ni^II^ atom are in the range of 1.829–1.936 Å (Fig. 1, Table 1), and the Ni–O distance shorter than that of Ni–N. The four atoms are coplanar with mean plane deviation of 0.027 (4) Å.

### Experimental

All the solvents and chemicals were of analytical grade and used without further purification. 2,6-Diformyl-4-methylphenol was prepared according to the literature method (Mandal *et al.* 1989; Long *et al.* 1983). N^1^-(2-aminoethyl)- N^2^-(4-nitrobenzyl)ethane-1,2-diamine was prepared according to the literature method (Jian *et al.* 2004; Hu *et al.* 2011). *L^2^* (0.119 g, 0.5 mmol) dissolved in 10 mL water was added dropwise to a solution of *L^1^* (0.082 g, 0.5 mmol) and Ni(ClO_4_)_2_.6H_2_O (0.183g, 0.5 mmol) in anhydrous ethanol (25 mL), the mixture was stirred at ambient temperature for 8 h and filtered. The orange block crystals suitable for the X-ray measurement were obtained by evaporation of the filtrate at room temperature for three weeks.

### Refinement

All H atoms for C-H distances were placed in calculated positions and included in the refinement in the riding-model approximation, with U(H) set to -1.2Ueq of the parent atom.

### Crystal data

**Table:**

[Ni(C_20_H_23_N_4_O_4_)]ClO_4_	*Z* = 2
*M**_r_* = 541.58	*F*(000) = 560
Triclinic, *P*1	*D*_x_ = 1.609 Mg m^−^^3^
Hall symbol: -P 1	Mo *K*α radiation, λ = 0.71073 Å
*a* = 9.5240 (14) Å	Cell parameters from 3108 reflections
*b* = 9.6423 (14) Å	θ = 2.3–28.0°
*c* = 14.024 (2) Å	µ = 1.04 mm^−^^1^
α = 97.897 (2)°	*T* = 291 K
β = 109.415 (3)°	Block, orange
γ = 107.453 (2)°	0.28 × 0.24 × 0.22 mm
*V* = 1117.7 (3) Å^3^	

### Data collection

**Table:**

Bruker SMART APEX CCD diffractometer	4314 independent reflections
Radiation source: sealed tube	3004 reflections with *I* > 2σ(*I*)
Graphite monochromator	*R*_int_ = 0.028
phi and ω scans	θ_max_ = 26.0°, θ_min_ = 2.3°
Absorption correction: multi-scan (*SADABS*; Bruker, 2000)	*h* = −11→11
*T*_min_ = 0.739, *T*_max_ = 0.786	*k* = −9→11
6276 measured reflections	*l* = −17→15

### Refinement

**Table:**

Refinement on *F*^2^	Primary atom site location: structure-invariant direct methods
Least-squares matrix: full	Secondary atom site location: difference Fourier map
*R*[*F*^2^ > 2σ(*F*^2^)] = 0.060	Hydrogen site location: inferred from neighbouring sites
*wR*(*F*^2^) = 0.138	H-atom parameters constrained
*S* = 1.10	*w* = 1/[σ^2^(*F*_o_^2^) + (0.0689*P*)^2^ + 0.0812*P*] where *P* = (*F*_o_^2^ + 2*F*_c_^2^)/3
4314 reflections	(Δ/σ)_max_ < 0.001
308 parameters	Δρ_max_ = 0.49 e Å^−^^3^
0 restraints	Δρ_min_ = −0.44 e Å^−^^3^

### Special details

**Table:**

Geometry. All esds (except the esd in the dihedral angle between two l.s. planes) are estimated using the full covariance matrix. The cell esds are taken into account individually in the estimation of esds in distances, angles and torsion angles; correlations between esds in cell parameters are only used when they are defined by crystal symmetry. An approximate (isotropic) treatment of cell esds is used for estimating esds involving l.s. planes.
Refinement. Refinement of F^2^ against ALL reflections. The weighted R-factor wR and goodness of fit S are based on F^2^, conventional R-factors R are based on F, with F set to zero for negative F^2^. The threshold expression of F^2^ > 2sigma(F^2^) is used only for calculating R-factors(gt) etc. and is not relevant to the choice of reflections for refinement. R-factors based on F^2^ are statistically about twice as large as those based on F, and R- factors based on ALL data will be even larger.

### Fractional atomic coordinates and isotropic or equivalent isotropic displacement parameters (Å^2^)

**Table:**

	*x*	*y*	*z*	*U*_iso_*/*U*_eq_	
C1	0.2128 (6)	0.2151 (5)	0.6715 (3)	0.0490 (11)	
H1	0.3112	0.2616	0.7281	0.059*	
C2	0.1781 (4)	0.2941 (4)	0.5895 (3)	0.0349 (8)	
C3	0.0262 (4)	0.2311 (4)	0.5055 (3)	0.0361 (8)	
H3	−0.0489	0.1421	0.5039	0.043*	
C4	−0.0125 (4)	0.3010 (4)	0.4249 (3)	0.0371 (8)	
C5	0.1016 (5)	0.4324 (5)	0.4308 (3)	0.0474 (10)	
H5	0.0762	0.4798	0.3776	0.057*	
C6	0.2522 (5)	0.4988 (4)	0.5112 (3)	0.0370 (8)	
C7	0.2919 (4)	0.4294 (4)	0.5956 (3)	0.0328 (8)	
C8	0.3605 (5)	0.6361 (5)	0.5105 (3)	0.0444 (9)	
H8	0.3318	0.6709	0.4510	0.053*	
C9	0.5962 (5)	0.8644 (5)	0.5798 (3)	0.0418 (9)	
H9A	0.5653	0.9445	0.6055	0.050*	
H9B	0.5828	0.8606	0.5077	0.050*	
C10	0.7678 (5)	0.8907 (5)	0.6473 (3)	0.0456 (10)	
H10A	0.8073	0.8262	0.6128	0.055*	
H10B	0.8365	0.9949	0.6616	0.055*	
C11	0.9105 (5)	0.8406 (5)	0.8155 (3)	0.0472 (10)	
H11A	0.9946	0.9394	0.8498	0.057*	
H11B	0.9477	0.7792	0.7764	0.057*	
C12	0.8651 (5)	0.7671 (4)	0.8951 (3)	0.0400 (9)	
H12A	0.9505	0.7381	0.9362	0.048*	
H12B	0.8471	0.8368	0.9422	0.048*	
C13	0.6434 (4)	0.5655 (4)	0.9063 (3)	0.0353 (8)	
H13A	0.5598	0.4680	0.8666	0.042*	
H13B	0.7246	0.5495	0.9624	0.042*	
C14	0.5743 (4)	0.6609 (4)	0.9526 (3)	0.0312 (7)	
C15	0.6606 (5)	0.7570 (4)	1.0532 (3)	0.0390 (9)	
H15	0.7632	0.7612	1.0912	0.047*	
C16	0.5967 (5)	0.8476 (5)	1.0984 (3)	0.0509 (11)	
H16	0.6567	0.9150	1.1644	0.061*	
C17	0.4424 (4)	0.8333 (4)	1.0419 (3)	0.0341 (8)	
C18	0.3494 (5)	0.7342 (5)	0.9422 (3)	0.0424 (9)	
H18	0.2446	0.7261	0.9062	0.051*	
C19	0.4163 (5)	0.6489 (5)	0.8986 (3)	0.0496 (11)	
H19	0.3560	0.5825	0.8323	0.060*	
C20	−0.1760 (5)	0.2319 (5)	0.3341 (3)	0.0455 (10)	
H20A	−0.2257	0.3053	0.3274	0.068*	
H20B	−0.2422	0.1461	0.3472	0.068*	
H20C	−0.1634	0.2008	0.2704	0.068*	
Cl1	0.88876 (12)	0.32607 (11)	0.79500 (8)	0.0463 (3)	
N2	0.4978 (4)	0.7171 (3)	0.5876 (3)	0.0393 (7)	
N3	0.7633 (3)	0.8528 (3)	0.7458 (2)	0.0307 (6)	
H3A	0.7438	0.9259	0.7817	0.037*	
N4	0.7161 (4)	0.6317 (4)	0.8366 (3)	0.0463 (8)	
H4	0.7513	0.5615	0.8131	0.056*	
N5	0.3722 (4)	0.9276 (3)	1.0862 (3)	0.0393 (7)	
Ni1	0.58985 (5)	0.66844 (6)	0.70964 (4)	0.03821 (17)	
O1	0.4300 (3)	0.4860 (3)	0.6787 (2)	0.0460 (7)	
O2	0.1258 (3)	0.0957 (3)	0.6725 (2)	0.0457 (7)	
O3	0.4487 (4)	1.0016 (4)	1.1791 (2)	0.0542 (8)	
O4	0.2448 (3)	0.9331 (3)	1.0331 (2)	0.0458 (7)	
O11	0.9790 (4)	0.4623 (4)	0.8000 (3)	0.0642 (10)	
O12	0.8093 (4)	0.2515 (3)	0.6896 (2)	0.0553 (8)	
O13	0.9309 (4)	0.2177 (3)	0.8403 (2)	0.0494 (7)	
O14	0.7706 (4)	0.3386 (3)	0.8214 (3)	0.0527 (8)	

### Atomic displacement parameters (Å^2^)

**Table:**

	*U*^11^	*U*^22^	*U*^33^	*U*^12^	*U*^13^	*U*^23^
C1	0.057 (3)	0.034 (2)	0.0290 (19)	−0.0028 (19)	0.0020 (18)	0.0108 (16)
C2	0.0257 (16)	0.0219 (16)	0.0395 (19)	0.0004 (13)	0.0019 (14)	0.0016 (14)
C3	0.0340 (19)	0.0333 (18)	0.0306 (18)	0.0112 (16)	0.0044 (15)	0.0006 (15)
C4	0.0330 (18)	0.041 (2)	0.0288 (17)	0.0206 (16)	0.0001 (14)	−0.0013 (15)
C5	0.039 (2)	0.048 (2)	0.035 (2)	0.0113 (18)	−0.0043 (17)	0.0095 (17)
C6	0.0396 (19)	0.0278 (17)	0.042 (2)	0.0162 (15)	0.0107 (16)	0.0097 (15)
C7	0.0268 (16)	0.0225 (16)	0.0392 (19)	0.0095 (14)	0.0007 (14)	0.0084 (14)
C8	0.046 (2)	0.038 (2)	0.043 (2)	0.0146 (18)	0.0089 (18)	0.0186 (17)
C9	0.045 (2)	0.040 (2)	0.036 (2)	0.0135 (17)	0.0109 (17)	0.0121 (16)
C10	0.051 (2)	0.037 (2)	0.042 (2)	0.0060 (18)	0.0174 (19)	0.0155 (18)
C11	0.041 (2)	0.043 (2)	0.047 (2)	0.0061 (18)	0.0127 (18)	0.0121 (18)
C12	0.0399 (19)	0.0326 (18)	0.0293 (18)	0.0033 (16)	0.0019 (15)	0.0061 (14)
C13	0.0355 (19)	0.0280 (17)	0.042 (2)	0.0137 (15)	0.0111 (16)	0.0147 (15)
C14	0.0332 (18)	0.0202 (15)	0.0344 (18)	0.0082 (14)	0.0076 (14)	0.0083 (13)
C15	0.046 (2)	0.0255 (18)	0.0349 (19)	0.0162 (16)	0.0035 (17)	0.0025 (14)
C16	0.049 (2)	0.048 (2)	0.037 (2)	0.014 (2)	0.0058 (18)	−0.0099 (18)
C17	0.0376 (19)	0.0390 (19)	0.0334 (19)	0.0109 (16)	0.0239 (16)	0.0147 (15)
C18	0.037 (2)	0.049 (2)	0.037 (2)	0.0136 (18)	0.0078 (16)	0.0194 (17)
C19	0.039 (2)	0.042 (2)	0.038 (2)	0.0032 (18)	−0.0005 (17)	−0.0135 (17)
C20	0.040 (2)	0.046 (2)	0.035 (2)	0.0166 (19)	0.0017 (17)	−0.0023 (17)
Cl1	0.0463 (6)	0.0431 (6)	0.0465 (6)	0.0159 (4)	0.0142 (4)	0.0146 (4)
N2	0.0400 (17)	0.0305 (16)	0.0445 (18)	0.0154 (14)	0.0107 (14)	0.0117 (13)
N3	0.0364 (16)	0.0258 (14)	0.0349 (16)	0.0115 (12)	0.0196 (13)	0.0092 (12)
N4	0.0458 (19)	0.0414 (18)	0.0373 (18)	0.0090 (16)	0.0078 (15)	0.0043 (14)
N5	0.0464 (18)	0.0294 (15)	0.0393 (18)	0.0135 (14)	0.0147 (15)	0.0071 (13)
Ni1	0.0278 (3)	0.0389 (3)	0.0393 (3)	0.0069 (2)	0.0085 (2)	0.0086 (2)
O1	0.0350 (14)	0.0392 (15)	0.0425 (16)	0.0036 (12)	−0.0002 (12)	0.0087 (12)
O2	0.0385 (15)	0.0402 (15)	0.0300 (13)	−0.0050 (12)	−0.0028 (11)	0.0086 (11)
O3	0.0502 (17)	0.0526 (18)	0.0440 (17)	0.0139 (14)	0.0136 (14)	−0.0118 (14)
O4	0.0422 (15)	0.0493 (17)	0.0461 (16)	0.0191 (13)	0.0152 (13)	0.0142 (13)
O11	0.056 (2)	0.055 (2)	0.0561 (19)	−0.0056 (16)	0.0158 (16)	0.0115 (15)
O12	0.0471 (16)	0.0429 (16)	0.0541 (18)	0.0146 (13)	−0.0071 (14)	0.0198 (14)
O13	0.0488 (16)	0.0480 (17)	0.0433 (15)	0.0168 (13)	0.0077 (13)	0.0165 (13)
O14	0.0452 (16)	0.0505 (17)	0.063 (2)	0.0222 (14)	0.0182 (15)	0.0161 (15)

### Geometric parameters (Å, º)

**Table:**

C1—O2	1.205 (5)	C13—C14	1.478 (5)
C1—C2	1.466 (5)	C13—N4	1.483 (5)
C1—H1	0.9300	C13—H13A	0.9700
C2—C7	1.394 (5)	C13—H13B	0.9700
C2—C3	1.411 (5)	C14—C15	1.392 (5)
C3—C4	1.397 (5)	C14—C19	1.402 (5)
C3—H3	0.9300	C15—C16	1.400 (6)
C4—C5	1.370 (6)	C15—H15	0.9300
C4—C20	1.522 (5)	C16—C17	1.372 (6)
C5—C6	1.384 (5)	C16—H16	0.9300
C5—H5	0.9300	C17—C18	1.398 (5)
C6—C8	1.417 (6)	C17—N5	1.465 (5)
C6—C7	1.437 (5)	C18—C19	1.376 (6)
C7—O1	1.327 (4)	C18—H18	0.9300
C8—N2	1.305 (5)	C19—H19	0.9300
C8—H8	0.9300	C20—H20A	0.9600
C9—N2	1.489 (5)	C20—H20B	0.9600
C9—C10	1.513 (6)	C20—H20C	0.9600
C9—H9A	0.9700	Cl1—O11	1.314 (3)
C9—H9B	0.9700	Cl1—O14	1.328 (3)
C10—N3	1.487 (5)	Cl1—O12	1.383 (3)
C10—H10A	0.9700	Cl1—O13	1.386 (3)
C10—H10B	0.9700	N2—Ni1	1.841 (3)
C11—N3	1.468 (5)	N3—Ni1	1.893 (3)
C11—C12	1.513 (6)	N3—H3A	0.9100
C11—H11A	0.9700	N4—Ni1	1.936 (4)
C11—H11B	0.9700	N4—H4	0.9100
C12—N4	1.487 (5)	N5—O4	1.216 (4)
C12—H12A	0.9700	N5—O3	1.241 (4)
C12—H12B	0.9700	Ni1—O1	1.829 (3)
			
O2—C1—C2	126.2 (4)	C15—C14—C13	120.8 (3)
O2—C1—H1	116.9	C19—C14—C13	120.4 (3)
C2—C1—H1	116.9	C14—C15—C16	121.5 (4)
C7—C2—C3	121.0 (3)	C14—C15—H15	119.2
C7—C2—C1	120.5 (3)	C16—C15—H15	119.2
C3—C2—C1	118.4 (3)	C17—C16—C15	117.5 (4)
C4—C3—C2	120.7 (3)	C17—C16—H16	121.2
C4—C3—H3	119.6	C15—C16—H16	121.2
C2—C3—H3	119.6	C16—C17—C18	122.8 (4)
C5—C4—C3	117.6 (3)	C16—C17—N5	119.0 (3)
C5—C4—C20	121.9 (3)	C18—C17—N5	118.1 (3)
C3—C4—C20	120.5 (4)	C19—C18—C17	118.5 (4)
C4—C5—C6	124.0 (4)	C19—C18—H18	120.7
C4—C5—H5	118.0	C17—C18—H18	120.8
C6—C5—H5	118.0	C18—C19—C14	120.9 (4)
C5—C6—C8	119.4 (4)	C18—C19—H19	119.6
C5—C6—C7	118.8 (3)	C14—C19—H19	119.6
C8—C6—C7	121.7 (3)	C4—C20—H20A	109.5
O1—C7—C2	118.2 (3)	C4—C20—H20B	109.5
O1—C7—C6	124.0 (3)	H20A—C20—H20B	109.5
C2—C7—C6	117.8 (3)	C4—C20—H20C	109.5
N2—C8—C6	124.6 (4)	H20A—C20—H20C	109.5
N2—C8—H8	117.7	H20B—C20—H20C	109.5
C6—C8—H8	117.7	O11—Cl1—O14	107.3 (2)
N2—C9—C10	105.6 (3)	O11—Cl1—O12	106.0 (2)
N2—C9—H9A	110.6	O14—Cl1—O12	102.5 (2)
C10—C9—H9A	110.6	O11—Cl1—O13	129.9 (2)
N2—C9—H9B	110.6	O14—Cl1—O13	105.0 (2)
C10—C9—H9B	110.6	O12—Cl1—O13	103.12 (18)
H9A—C9—H9B	108.8	C8—N2—C9	119.4 (3)
N3—C10—C9	105.2 (3)	C8—N2—Ni1	126.6 (3)
N3—C10—H10A	110.7	C9—N2—Ni1	114.0 (2)
C9—C10—H10A	110.7	C11—N3—C10	115.1 (3)
N3—C10—H10B	110.7	C11—N3—Ni1	109.1 (2)
C9—C10—H10B	110.7	C10—N3—Ni1	108.2 (2)
H10A—C10—H10B	108.8	C11—N3—H3A	108.1
N3—C11—C12	105.3 (3)	C10—N3—H3A	108.1
N3—C11—H11A	110.7	Ni1—N3—H3A	108.1
C12—C11—H11A	110.7	C13—N4—C12	112.6 (3)
N3—C11—H11B	110.7	C13—N4—Ni1	121.8 (3)
C12—C11—H11B	110.7	C12—N4—Ni1	109.0 (3)
H11A—C11—H11B	108.8	C13—N4—H4	103.8
N4—C12—C11	107.6 (3)	C12—N4—H4	103.8
N4—C12—H12A	110.2	Ni1—N4—H4	103.8
C11—C12—H12A	110.2	O4—N5—O3	122.0 (3)
N4—C12—H12B	110.2	O4—N5—C17	120.8 (3)
C11—C12—H12B	110.2	O3—N5—C17	117.2 (3)
H12A—C12—H12B	108.5	O1—Ni1—N2	96.40 (13)
C14—C13—N4	113.3 (3)	O1—Ni1—N3	176.88 (13)
C14—C13—H13A	108.9	N2—Ni1—N3	86.25 (13)
N4—C13—H13A	108.9	O1—Ni1—N4	90.39 (14)
C14—C13—H13B	108.9	N2—Ni1—N4	171.64 (15)
N4—C13—H13B	108.9	N3—Ni1—N4	86.84 (14)
H13A—C13—H13B	107.7	C7—O1—Ni1	126.2 (2)
C15—C14—C19	118.7 (4)		
			
O2—C1—C2—C7	−177.4 (5)	C13—C14—C19—C18	178.2 (4)
O2—C1—C2—C3	3.6 (7)	C6—C8—N2—C9	−176.1 (4)
C7—C2—C3—C4	1.6 (6)	C6—C8—N2—Ni1	4.6 (7)
C1—C2—C3—C4	−179.4 (4)	C10—C9—N2—C8	−153.0 (4)
C2—C3—C4—C5	−0.4 (6)	C10—C9—N2—Ni1	26.4 (4)
C2—C3—C4—C20	179.6 (4)	C12—C11—N3—C10	−167.1 (3)
C3—C4—C5—C6	0.5 (6)	C12—C11—N3—Ni1	−45.2 (4)
C20—C4—C5—C6	−179.4 (4)	C9—C10—N3—C11	168.3 (3)
C4—C5—C6—C8	−179.1 (4)	C9—C10—N3—Ni1	46.0 (3)
C4—C5—C6—C7	−1.8 (7)	C14—C13—N4—C12	69.5 (4)
C3—C2—C7—O1	177.3 (3)	C14—C13—N4—Ni1	−62.8 (4)
C1—C2—C7—O1	−1.7 (6)	C11—C12—N4—C13	−169.7 (3)
C3—C2—C7—C6	−2.8 (6)	C11—C12—N4—Ni1	−31.3 (4)
C1—C2—C7—C6	178.2 (4)	C16—C17—N5—O4	169.9 (4)
C5—C6—C7—O1	−177.2 (4)	C18—C17—N5—O4	−8.8 (5)
C8—C6—C7—O1	0.1 (6)	C16—C17—N5—O3	−9.5 (5)
C5—C6—C7—C2	2.9 (6)	C18—C17—N5—O3	171.8 (3)
C8—C6—C7—C2	−179.8 (4)	C8—N2—Ni1—O1	0.3 (4)
C5—C6—C8—N2	171.9 (4)	C9—N2—Ni1—O1	−179.0 (3)
C7—C6—C8—N2	−5.4 (7)	C8—N2—Ni1—N3	178.7 (4)
N2—C9—C10—N3	−45.3 (4)	C9—N2—Ni1—N3	−0.6 (3)
N3—C11—C12—N4	49.4 (4)	C11—N3—Ni1—N2	−152.1 (3)
N4—C13—C14—C15	−97.3 (4)	C10—N3—Ni1—N2	−26.2 (3)
N4—C13—C14—C19	86.9 (4)	C11—N3—Ni1—N4	23.2 (3)
C19—C14—C15—C16	−3.8 (6)	C10—N3—Ni1—N4	149.1 (3)
C13—C14—C15—C16	−179.7 (4)	C13—N4—Ni1—O1	−42.5 (3)
C14—C15—C16—C17	3.0 (6)	C12—N4—Ni1—O1	−176.3 (3)
C15—C16—C17—C18	−0.6 (6)	C13—N4—Ni1—N3	138.9 (3)
C15—C16—C17—N5	−179.3 (4)	C12—N4—Ni1—N3	5.1 (3)
C16—C17—C18—C19	−0.8 (6)	C2—C7—O1—Ni1	−174.5 (3)
N5—C17—C18—C19	177.8 (4)	C6—C7—O1—Ni1	5.7 (6)
C17—C18—C19—C14	0.0 (6)	N2—Ni1—O1—C7	−5.3 (4)
C15—C14—C19—C18	2.3 (6)	N4—Ni1—O1—C7	179.6 (3)
